# Comparison and Phylogenetic Analysis of Mitochondrial Genomes of Talpidae Animals

**DOI:** 10.3390/ani13020186

**Published:** 2023-01-04

**Authors:** Di Xu, Mengyao Sun, Zenghao Gao, Yiping Zhou, Qingqian Wang, Lei Chen

**Affiliations:** College of Life Sciences, Qufu Normal University, Qufu 273165, China

**Keywords:** Talpidae, mitochondrial genome, phylogenetic, ecotype

## Abstract

**Simple Summary:**

Talpidae animals are commonly used in ecological studies due to their diverse ecotypes. In this study, by comparing the mitochondrial genomes of 14 species belonging to the family Talpidae, it is found that the variation of repeats in the control region is the main reason for the difference in the length of Talpidae mitochondrial genomes. By using the mitochondrial genome *Cyt b* genes of 48 species in family Talpidae as biomarkers, the phylogenetic tree of Talpidae was reconstructed. By combining the divergence time of Talpidae animals with the changes in the geological history period, it is found that the divergence of Talpidae is closely related to historically global climate changes. These results provide useful experimental data for systematic evolution of Talpidae, and also provide the basis for the study of ecological adaptability of Talpidae.

**Abstract:**

Talpidae is a model group for evolutionary studies due to their highly specialized morphologies and diverse lifestyles. Mitochondrial genomes are molecular markers commonly used in species evolution and phylogenetic studies. In this study, the complete mitochondrial genome sequence of *Scaptochirus moschatus* was obtained by Illumina NovaSeq sequencing. The complete mitochondrial genomes of 14 Talpidae species (including *Scaptochirus moschatus* obtained in the present study) and the cytochrome b (*Cyt b*) gene sequences of 48 Talpidae species were downloaded from the NCBI database for comparison and phylogenetic studies to analyze the phylogenetic relationships and to find the possible reasons of the niche differentiation and ecotype specialization of Talpidae animals. The results showed that the mitochondrial genome sequences of 14 species belonging to the family Talpidae were 16,528 to 16,962 bp, all containing 13 protein-coding genes, 22 tRNA, two rRNA, and a non-coding region (control region). The difference in the number of repetitive repeats in the control region is responsible for the difference in the length of Talpidae mitochondrial genome sequences. Combining the divergence time of Talpidae animals with the geological history, it is found that the niche differentiation and ecotype divergence of Talpidae is closely related to historically global climate changes. Semi-aquatic groups diverged in the early Oligocene (about 31.22 MYA), probably in response to the global climate transition from warm to cool. During the early Miocene (about 19.54 MYA), some species of Talpidae moved to underground habitats and formed fossorial groups that were adept at digging due to the effects of the glaciation. In the middle Miocene (about 16.23 MYA), some Talpidae animals returned to the ground and formed semi-fossorial shrew moles as global climate warming again.

## 1. Introduction

Talpidae, a family of insectivores widespread in Asia, Europe, and North America, originated from the old world and spread from Eurasia to North America [1]. Talpidae animals are commonly used in ecological studies due to their diverse ecotypes. Most Talpidae animals live in semi-underground or underground burrowing. A few of them live on the ground or are semi-aquatic animals [2]. They are considered to be a good model for the study of mammalian adaptive evolution also because of their adaptive morphological features and sensory system in different habitats [3,4]. The morphological characteristics of Talpidae animals, such as skeletal morphology [5,6], muscle structure [7], cortical organization [8], and sexual organs [9] have been reported, but their morphological diversity is much higher than the current cognition.

The phylogenetic status of Talpidae was discussed based on morphological and mitochondrial gene sequence analysis, and it is believed that Talpidae is closely related to the family Soricidae [1,10]. Recently, the phylogenetic relationships of Talpidae animals have attracted much attention from biologists. Whidden and Sánchez-Villagra et al. preliminarily explored the evolutionary relationship of Talpidae animals from the perspective of morphology [1,11]. Shinohara et al. found that the North American moles and Eurasian moles are not monophyletic groups, and thus proposed that there have been at least two separate evolution processes of Talpidae [12]. He et al. confirmed that Gansu mole (*Scapanulus oweni*) is the only living Asian population in North America based on mitochondrial and nuclear gene sequences [2]. However, with the reported new species belonging to the family Talpidae, such as *Talpa aquitania*, *Uropsilus dabieshanensis sp. nov*., etc. [13,14], the phylogenetic relationships of Talpidae need to be further explored.

Mitochondrial genomes as extracellular genetic material are widely used in phylogenetic studies because of their small molecular size, maternal inheritance, rapid evolution rate, and absence of recombination [15]. In particular, the cytochrome b (*Cyt b*) gene in the mitochondrial genome is widely used in mammalian phylogenetic studies as a barcode gene [16,17]. According to the previous reports, the mitochondrial genome of Talpidae is a circular DNA molecule containing 13 protein-coding genes, 22 tRNA, two rRNA and a control region [18,19]. The *Scaptochirus moschatus* is endemic to China. In this study, firstly, Illumina NovaSeq sequencing was performed to obtain the complete mitochondrial genome of the *S. moschatus*. Then, by comparing the mitochondrial genome of *S. moschatus* with the existing mitochondrial genome data of other Talpidae animals in the NCBI database, the mitochondrial genome composition and structural differences of moles were analyzed to find key information on genetic differences among species. Thirdly, the mitochondrial genome *Cyt b* genes were used as biomarkers to reconstruct the phylogenetic trees of Talpidae, and their divergence time was estimated by using the molecular clock hypothesis. Combined with the relevant geological historical events, the possible reasons for the ecotype divergence of Talpidae animals will be discussed to provide scientific data for the studies of the systematic evolution and ecological adaptation of Talpidae.

## 2. Materials and Methods

### 2.1. Sample Collection

Female *S. moschatus* was collected from Liaocheng city, Shandong Province, China. No harm was caused to the animal or to its habitat during the sampling process. The animal was transported back to the laboratory without injury, and *S. moschatus* was euthanized according to the Laboratory Animal Anaesthesia method recommended by the Association for Assessment and Accreditation of Laboratory Animal Care (AAALAC) requirements. Then, the hind-limb musculature was harvested for genomic DNA extraction and sequencing.

This study was conducted in accordance with the regulations of the Bioethics Committee of Qufu Normal University (No. 2022073), and met the requirements of the China Wildlife Conservation Association and Chinese laws.

### 2.2. DNA Extraction, PCR Amplification and Sequencing

Total DNA was extracted using E.Z.N.A^®^ Tissue DNA kit (OMEGA, Biel/Bienne, Switzerland). Bridging PCR amplification based on the cBot solid phase vector and Illumina NovaSeq sequencing with the read length of 2 × 150 bp were performed to get the mitochondrial genome sequence of *S. moschatus*. MITOS software was used to predict protein encoding, tRNA, and rRNA genes in the mitochondrial genome. The start and end codon positions of mitochondrial genes were manually corrected. The CGView software was used to display the mitochondrial genome.

### 2.3. Comparative Analysis of Mitochondrial Genomes of Talpidae Animals

The mitochondrial genome sequences of 13 species in 7 genera of Talpidae were downloaded from NCBI (Table S1). MEGA X software was used for comparative analysis of the mitochondrial genomes of Talpidae animals [20]. A new alignment was built by ALIGN in the MEGA X software. All the sequences were aligned by using the ClustalW algorithm. Base content and codon usage were counted. The codon usage was visually displayed through Origin 2021 software (OriginLab Corporation, Northampton, MA, USA). The tRNA structures of 14 Talpidae animals were predicted by using tRNAscan-SE software [21].

The control region of the mitochondrial genome of 14 species of Talpidae animals was analyzed and compared by Bioedit [22]. The Tandem Repeats Finder Program was used to search for tandem repeats in the control region [23]. According to the regional division method of the mammalian mitochondrial genome control region, the conserved and tandem repetition sequences of the control region of the mitochondrial genome of Talpidae animals were searched and labeled.

### 2.4. Phylogenetic Analysis of Talpidae Animals

The *Cyt b* genes of 48 species belonging to the family Talpidae were downloaded from NCBI were used for phylogenetic analysis (Table S2). *Erinaceus amurensis* (KX964606.1) was used as an outgroup. Maximum Likelihood (ML), Neighbor-joining (NJ) and Minimum evolution (ME) methods were used to construct phylogenetic trees for phylogenetic analysis. The Bootstrap value was set to 1000. We calculated the corrected genetic distance for the *Cyt b* gene sequences of these 48 species with the Model parameter set to Kimura 2-parameter and the Substitutions to Include option set to d: Transtions + Transversions. The phylogenetic tree was constructed, and the genetic distance was calculated using MEGA X software [20]. The ML tree based on the *Cyt b* gene was used to predict the differentiation time of Talpidae animals using the CLOCKS function in MEGA X, with the calibration type selected as Min Time Only and Max Time Only. The divergence time of *Euroscaptor* and *Mogera* was selected as the calibration point in Min Time Only, and the divergence time of *Uropsilus* and other genera was selected as the calibration point in Max Time Only. The divergence time was obtained by getting the divergence time in the Time Tree [24].

## 3. Results

### 3.1. Mitochondrial Genome Composition of S. moschatus

**Figure 1:**
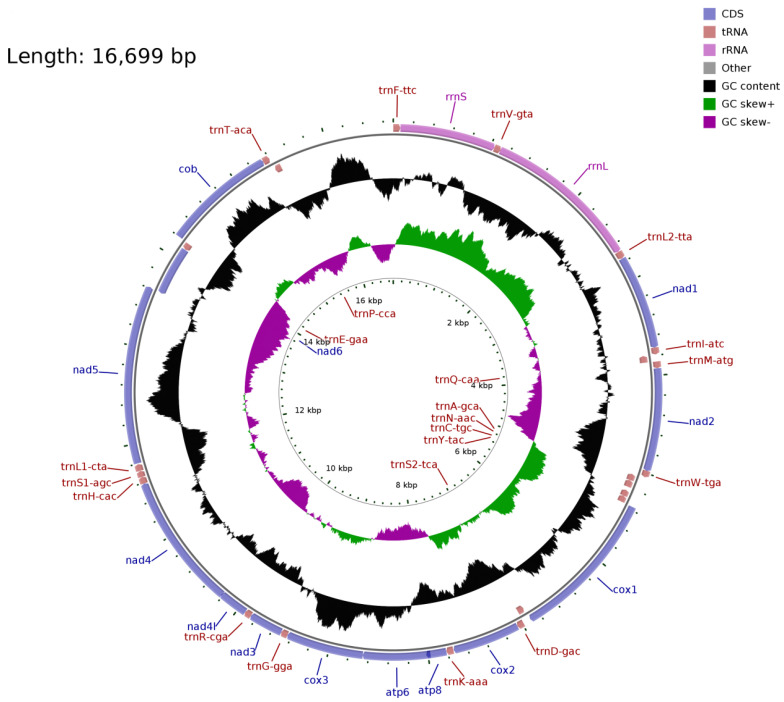
The mitochondrial genome of the short-faced mole (*Scaptochirus moschatus*).

The mitochondrial genome of *S. moschatus* is consistent with that of reported Talpidae animals. The *tRNA-Ile*, *tRNA-Ala*, *tRNA-Cys*, *tRNA-Asn*, *tRNA-Tyr*, *tRNA-Ser*, *ND6*, *tRNA-Glu,* and *tRNA-Pro* genes are located on the L strand, and the other genes are located on the H strand. The composition and arrangement of the mitochondrial genome is consistent with other Talpidae animals (Figure 1). The total length of the 13 protein-coding genes is 11,397 bp, with the highest frequency of start codon ATG and stop codon TAA. Most tRNA can fold into typical clover secondary structures, except tRNA-Ser lacking a dihydrouracil ring. The mitochondrial whole-genome data for *S. moschatus* has been submitted to the NCBI GenBank with the accession number MZ594566.

### 3.2. Comparative Analysis of the Talpidae Mitochondrial Genomes

#### 3.2.1. Mitochondrial Genome Composition

The length of the mitochondrial genomes of Talpidae animals is 16,528 bp to 16,962 bp, with the same gene composition and arrangement (Table S1). The base content of Talpidae mitochondrial genomes is A > T > G > C, and the proportion of AT (59.9% to 73.4%) is higher than that of GC (35.6% to 40.0%). In addition, at most site of the codons, the abundance of A and T are significantly higher than that of G and C, indicating a significant preference of AT bases (Table 1 and Table 2).

A total of 17,218 sites are obtained from the alignment of Talpidae mitochondrial genomes, including 1007 conserved sites (6.3% of total sites), 5559 parsimony-informative sites (32.3%), 1448 singleton sites (8.4%), and 7011 variable sites (40.7%). In variant sites, there are 1664 transitional sites (accounting for 23.7% of variant sites), and 1182 transversional sites (accounting for 16.9% of variant sites). The ratio of transitions/transversions is 1.4.

#### 3.2.2. Protein-Coding Genes

**Figure 2:**
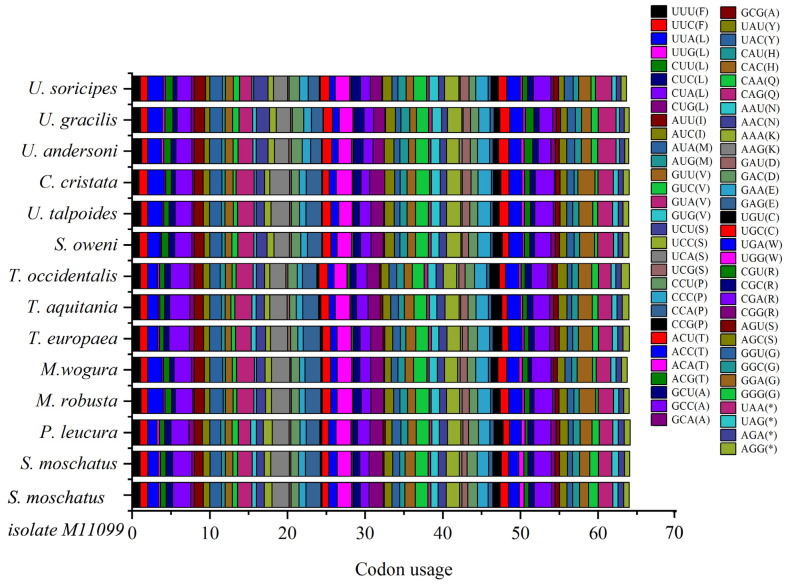
Codons usage of the Talpidae animals’ mitochondrial genomes. The codons CUA (L), UCA (S), CGA (R), GUA (V), UAA (*), ACA (T), and CCA (P) are used more frequently, and codon GCG (A) is less used.

Among the Talpidae mitochondrial protein-encoding genes, codons CUA (L), UCA (S), CGA (R), GUA (V), UAA (*), ACA (T), and CCA (P) are used more frequently, and codon GCG (A) is less used, especially in *Condylura cristata* (Figure 2, Table S3).

The start codon of Talpidae mitochondrial genes *ND1*, *COX1*, *COX2*, *ATP8*, *ATP6*, *COX3*, and *ND4L* is ATG. There are two species (*S. oweni*, *U. andersoni*) whose initiation codon of the *ND2* gene is ATT, and the initiation codon of the other species is ATA. The start codon of the *ND3* genes is ATA in three species (*U. andersoni*, *U. gracilis*, *U. soricipes*), while in other species it is ATT. In *S. oweni*, *U. soricipes* and *U. gracilis*, the start codon of the *ND4* gene is GTG, while the start codon of the *ND4* of the other eleven species is ATG. The start codon of the *ND5* gene is ATT for twelve Talpidae animals, but in *S. moschatus* and *S. oweni*, it is ATA. The start codon of the *ND6* gene is ATG in most Talpidae animals except for *P. leucura* (ATA) and *S. moschatus Isolate M11099* (TTA). In addition, only the *Cyt b* gene of *C. cristata* have a start codon of ATT. Other Talpidae animals’ *Cyt b* genes start with ATG.

TAA (TA-) is the most frequently used stop codon in Talpidae mitochondrial genes. Each animal has three to five incomplete stop codons, which are complemented to TAA by Poly A at the mRNA 3′ end. Most Talpidae animals’ *Cyt b* genes stop with AGA except *T. europaea*, which has an incomplete stop codon. The stop codons of the *ND2* genes in *M. wogura*, *T. europaea*, *U. talpoides*, the stop codons of the *COX1* genes in *U. andersoni*, *U. gracilis*, *U. soricipes*, and the stop codons of *ND1* gene in *U. talpoides* are TAG (Table 3).

#### 3.2.3. RNA Genes

**Figure 3:**
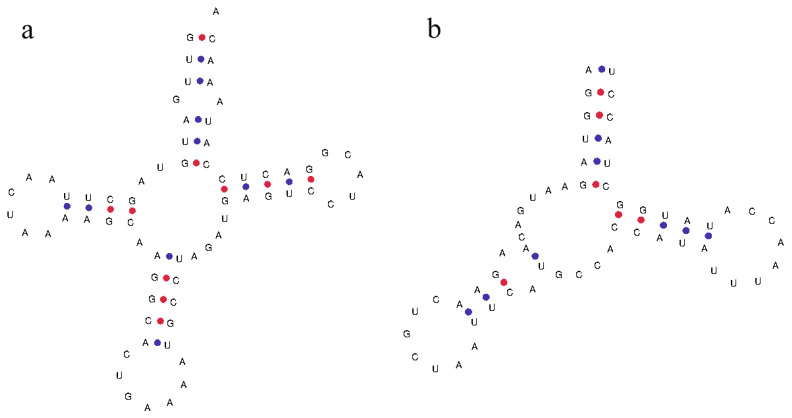
(**a**) Typical trefoil type secondary structure of *tRNA-Phe*. (**b**) Secondary structure of *tRNA-Ser*, which lack the dihydrouracil ring.

Talpidae mitochondrial 12S rRNA genes are located between the *tRNA-Phe* and the *tRNA-Val* genes, with a sequence length of 959 bp to 987 bp. The 16S rRNA genes are located between the *tRNA-Val* and the *tRNA-Leu* genes, with a sequence length of 1561 bp to 1589 bp. By predicting the secondary structure of the tRNA, it is found that most of the tRNA genes can conform to a typical clover structure, but some tRNA genes (such as the *tRNA-Ser* of *C. cristata*) lack the dihydrouracil ring (Figure 3).

#### 3.2.4. Analysis of the Control Region of the Mitochondrial Genome

The control region of the Talpidae animals lies after *tRNA-Pro*, with a sequence length of 1069 (*U. gracilis*) to 1504 bp (*T. occidentalis*). The control region of the genus *Uropsilus* is shorter than other Talpidae animals, especially *U. gracilis*, with the shortest sequence length of only 1069 bp. Partition alignment reveals that the composition of mitochondrial control region varies widely across species, with CSB-1 presenting in all animals, CSB-2 lacking in *S. oweni*, and CSB-2 and CSB-3 lacking in *U. gracilis* (Table 4, Figure S1).

Analysis of tandem repeats in mitochondrial genomes finds that tandem repeats are relatively rare in the genus *Uropsilus*, which are found only in *U. soricipes.* The repetitive sequence of *U. soricipes* is located at the sites from 19 bp to 52 bp, while tandem repeats of other species are mainly located at the sites from 720 to 1118 bp. The repeat unit of *U. soricipes* has only two bases (AT), while the repeat units of the other Talpidae animals contain more than eight bases. The sequence with the highest number of repeats is *T. occidentalis*, which occurs 39 times. Its repeat unit contains 10 bases (CACGTACGCA). The maximum copy number of tandem repeats makes the sequence length of the control region of *T. occidentalis* mitochondrial genome much higher than other Talpidae species (Table 5).

### 3.3. Phylogenetic Analysis of Talpidae

#### 3.3.1. Genetic Distance

To explore the phylogenetic relationships of the family Talpidae calculated corrected genetic distances were analyzed using MEGA X software. The results show that the genetic distance between Talpidae species is 0.00088 to 0.27661. Among them, the genetic distance between *Uropsilus nivatus* and *U. gracilis* is the smallest (0.00088). The genetic distance between *T. occidentalis* and *U. atronates* is the highest (0.27661) (Table S4).

#### 3.3.2. Phylogenetic Analysis

A total of 49 *Cyt b* gene sequences were involved in phylogenetic analysis. There are 1140 locations in the final dataset. The phylogenetic trees constructed by different methods all show that Talpidae animals are obviously divided into two clades. Eight species of the genus *Uropsilus* gather into a clade, and other genera cluster in the other clade. Comparison of the *Cyt b* genes of 48 species belonging to the family Talpidae showed that there is a C at site 241 and a T at position 592 in genus *Uropsilus*, which is different from other genera (Figure S2).

**Figure 4:**
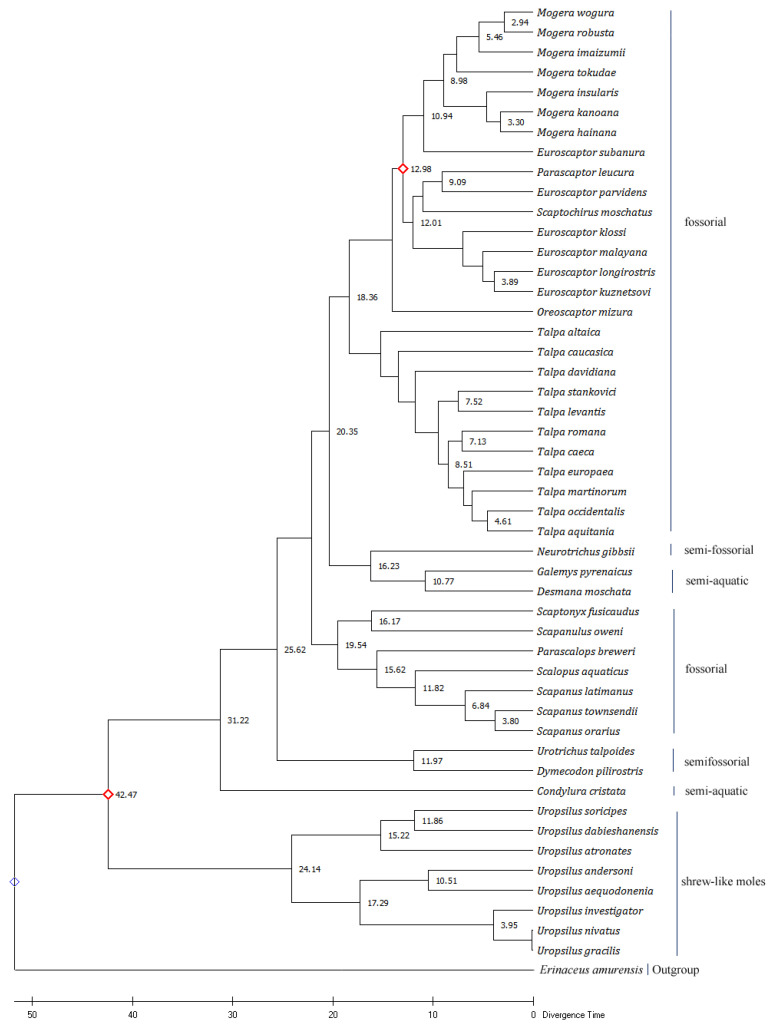
The Maximum Likelihood tree based on mitochondrial *Cyt b* genes of 48 species belonging to the family Talpidae. The Bootstrap value was set to 1000. The values on the evolutionary tree branch represent the divergence time (million years ago). The red diamonds are the calibration points chosen when predicting the molecular clock.

The topologies varied slightly among ML, NJ, and ME trees. On the ML tree, *N. gibbsii* is closely related to *Galemys pyrenaicus*, and *Desmana moschata* diverged from a common ancestor. *Oreoscaptor mizura* clusters into a single clade and is closely related to the genus *Euroscaptor*. *P. leucura* and *Euroscaptor parvidens* cluster in a clade, and then cluster with *S. moschatus* into a sister group. NJ tree shows that *Neurotrichus gibbsii* is closely related to the genus *Talpa*. *Talpa levantis*, *Talpa caeca*, and *Talpa stankovici* of the genus *Talpa* are grouped into a clade, and *Talpa romana* is separated into another clade. But in the ML phylogenetic tree, *T. stankovici* and *T. levantis* of the *Talpa* genus cluster into a clade, and *T. romana* and *T. caeca* cluster into another clade. *O. mizura* is closely related to the genus *Euroscaptor* in the ML tree, but the NJ tree shows that *P. leucura* is more closely related to *O. mizura* and *E. parvidens.* In the ME tree, *O. mizura* is clustered into a clade with *P. leucura*, *E. parvidens*, and *S. moschatus*. However, it is worth noting that all of the phylogenetic trees constructed based on the *Cyt b* gene show that *E. subanura* did not cluster with the other five species of *Euroscaptor*, but with the genus *Mogera*. In addition, *E. parvidens* is more closely related to *P. leucura* than other species from the same genus (Figure 4, Figures S3 and S4).

Calculations based on the ML tree found that the divergence time between *Uropsilus* and other genera was calculated at 42.47 million years ago (MYA). At Oligocene (about 31.22 MYA), *C. cristata* was isolated from other clades. The divergence time of *Urotrichus*, *Dymecodon* with other genera was about 25.62 MYA, and that of *Urotrichus* and *Dymecodon* was about 11.97 MYA. The divergence time between semi-aquatic moles (*Desmana*, *Galemys*) and semi-fossorial shrew moles (*Neurotrichus*) was about 16.23 MYA. The semi-aquatic moles (*Desmana*, *Galemys*), semi-fossorial shrew moles (*Neurotrichus*), and fossorial moles (*Talpa, Oreoscaptor*, *Euroscaptor*, *Parascaptor*, *Scaptochirus*, *Mogera*) differentiated at about 20.35 MYA. Thus, we conjectured that the earliest divergence time of Talpidae animals was about 42.47 MYA in the middle Eocene. The North American semi-aquatic *C. cristata* appeared at about 31.22 MYA during the Oligocene. Talpidae animals differentiated into two groups at about the late Oligocene (25.62 MYA), one of which evolved into semi-fossorial shrew moles (*Urotrichus*, *Dymecodon*) at about 11.97 MYA. At about 20.35 MYA, Talpidae animals underwent another niche distribution, when semi-aquatic and semi-fossorial moles appearing (Figure 4).

## 4. Discussion

As a genetic material outside of the nucleus, mitochondrial genomes are relatively small and highly conserved and are commonly used for phylogenic studies [25,26,27]. Thirteen mitochondrial genomes of Talpidae species have been reported, and the composition of the mitochondrial genomes is consistent with the general characteristics of mammals [28,29,30]. Among the initiation codons of protein-coding genes, ATG was used frequently with a frequency of more than 69%. TAA was used frequently as a stop codon. It was reported that the termination codon AGA was commonly used in mammals’ mitochondrial *Cyt b* gene [31,32]. In amphibians, reptiles, and birds, the stop codons of *Cyt b* genes were mostly terminated by T-, TA- and TAA [33,34,35]. The termination codons of *Cyt b* genes in most Talpidae animals conformed to the general characteristics of mammals. However, the *Cyt b* gene of *T. europaea* was found to have an incomplete stop codon (T-).

It was found that there were many repeated sequences in the control region, which was the region with the largest variation in sequence length in the mitochondrial genome [22,36,37]. The analysis of the tandem repeats in the control region of Talpidae animals revealed that the number of tandem repeats differs across species. Especially the repetitive sequence of *T. occidentalis*, which appeared 10 times and contained 379 bases in the repetitive region, resulting in a significantly longer mtDNA sequence of *T. occidentalis* than other species. The CSB domain is the short conservative sequence region present in the control region. Previous studies showed that the CSB-1 domain was highly conserved in most vertebrates, while some vertebrates lost the CSB-2 or CSB-3 domain [22,38]. In the present study, the CSB-1 domain was present in all Talpidae animals. However, there was no CSB-2 domain in *S. oweni*, and the CSB-2 and CSB-3 domains were missing in *U. gracilis*. These results indicated that domains CSB-2 and CSB-3 were not absolutely conserved, and these two domains may be variable during evolution absent in some species.

The phylogenetic relationship of Talpidae is a controversial topic in ecological research. He et al. used 19 nuclear genes and two mitochondrial genes to construct the phylogenetic trees of about 60% of Talpidae animals in 17 genera, preliminarily elucidating the evolutionary relationships of the Talpidae animals [2]. However, with the emergence of new species of Talpidae in recent years (e.g., *Talpa aquitania*, *Uropsilus dabieshanensis sp. nov.*), the phylogenetic relationship of Talpidae animals needs to be further updated. In this study, we reconstructed the phylogenetic trees of 48 species from 18 genera using *Cyt b* genes. It was found that the topologies of the phylogenetic trees constructed by different methods were different, but they all showed that the species of the same genus were closely related. The phylogenetic trees all showed that *Uropsilus* belonged to a monophyletic group, which was consistent with the results of previous studies [39]. The genus *Uropsilus* was the most primitive group of the family Talpidae. They inherited ancestral morphological features adapted to above-ground life, including underdeveloped forepaws and distinct external ears [14,40]. Thus, the genus *Uropsilus* is in the earlier clade of the mole family phylogeny. They occupy a separate niche. Mitochondrial genome comparisons revealed that the bases at sites 241 and 592 of the *Cyt b* gene of the genus *Uropsilus* are different from the other genera. The tandem repeats of the genus *Uropsilus* are significantly fewer than other Talpidae animals. We speculated that point mutations of bases and differences in tandem repeats within the control region might be one of the main variations in the mitochondrial genome during the evolution of the family Talpidae.

Morphological studies showed that *Scaptochirus* and *Parascaptor* were similar in the auditory region of the skull, especially in the middle ear [41,42]. Previous molecular studies also indicated that *S. moschatus* and *P. leucura* were sister species [19]. However, in the present study, the ML, ME, and NJ trees all showed that *P. leucura* clustered with *E. parvidens* first and then clustered into a clade with *S. moschatus*. This indicated that *P. leucura* was more closely related to *E. parvidens* than to *S. moschatus*. Previous studies found that *Euroscaptor subanura* was closely related to *E. parvidens* [43]. However, in the present study, the topology of the phylogenetic trees showed that *Euroscaptor subanurat* was separated from the genus *Euroscaptor* and clustered into the genus *Mogera*, while the other five species of the genus *Euroscaptor* clustered into one clade, which was different from the previous reports. The phylogenetic relationship between the genus *Euroscaptor* and the evolutionary status of *E. subanura* still needs to be further confirmed by morphological and other molecular studies.

The fluctuation of climate and the emergence of glaciation were always accompanied by the evolution of organisms and the formation of new species [44,45]. The Eocene (about 56 MYA-34 MYA) contained the warmest geological period of the past 65 million years [46,47]. The common ancestor of the Talpidae animals appeared in the middle Eocene (about 42.47 MYA). In the warm climate of the early and middle Eocene, the ancestors of Talpidae animals adapted to terrestrial life. Subsequently, global temperatures dropped significantly and ice sheets formed in Antarctica and expanded rapidly. In the early Oligocene (34 MYA to 30 MYA), the global climate became drier and cooler [48]. To adapt to the change in the global climate, the ancestors of Talpidae animals began to distribute closer to rivers and lakes, and the semi-aquatic *C. cristata* appeared in North America at about 31.22 MYA. *C. cristata* was the only species in the Talpidae family living in North America with a semi-aquatic habit that allows them to forage in the water during the cold season [49]. With global warming in the late Oligocene [50], Talpidae animals diverged into two groups at about 25.62 MYA. One of them evolved into semi-fossorial shrew moles (*Urotrichus*, *Dymecodon*) at about 11.97 MYA in the Miocene. A high glaciation occurred during the Oligocene and Miocene transition periods (about 23 MYA). Then there were a series of interspaced lower glaciations [45]. Due to the influence of the early Miocene glaciation, the fossorial Talpidae animals diverged at about 19.54 MYA. In the late Oligocene, climate warming kept the global ice volume at a low level until the middle Miocene (17 MYA -15 MYA). The semi-fossorial shrew moles (*Neurotrichus*) differentiated from other Talpidae animals at about 16.23 MYA. The above results show that the divergence time of different ecotypes of Talpidae animals basically coincides with the transition period of global climate during geological history. Therefore, historically global climate change may promote the niche differentiation and ecotype specialization of Talpidae animals. 

## 5. Conclusions

This paper compared the mitochondrial genomes of Talpidae animals, explored the phylogenetic relationships of Talpidae animals, and discussed the possible time and reasons for their ecotype differentiation by phylogenetic studies. Although all the mitochondrial genomes of Talpidae animals that have been obtained so far are involved, there are still a large number of mitochondrial genomes of Talpidae animals that have not been reported. Despite the great value of mitochondrial genomes in phylogenetic studies, a comprehensive analysis should still be combined with morphological traits, ecological habits, and other molecular markers to draw scientific conclusions.

## Figures and Tables

**Table 1:** The base content (%) of Talpidae animals’ mitochondrial genomes.

	T(U)	C	A	G	AT	CG
*Scaptochirus moschatus*	26.9	25.3	33.8	14.0	60.7	39.3
*Scaptochirus moschatus isolate M11099*	26.8	25.4	33.7	14.1	60.5	39.5
*Scapanulus oweni*	28.7	23.9	34.0	12.5	62.7	37.4
*Parascaptor leucura*	26.4	25.7	33.5	14.0	59.9	40.0
*Mogera robusta*	29.1	22.8	35.2	12.8	64.3	37.1
*Mogera wogura*	28.4	23.6	35.0	13.0	63.4	36.6
*Talpa europaea*	27.0	24.6	34.1	14.3	61.1	38.9
*Talpa aquitania*	26.8	24.8	34.2	14.2	61.0	39.0
*Talpa occidentalis*	26.6	24.9	34.1	14.4	60.7	39.3
*Condylura cristata*	28.6	23.0	35.6	12.8	64.2	35.8
*Urotrichus talpoides*	28.3	24.1	34.3	13.4	62.6	37.5
*Uropsilus andersoni*	30.1	22.9	33.2	13.8	63.3	36.7
*Uropsilus gracilis*	30.3	22.7	33.3	13.6	63.6	36.3
*Uropsilus soricipes*	30.5	22.4	33.9	13.2	64.4	35.6

**Table 2:** The base content (%) of Talpidae animals’ mitochondrial genomes at different sites of the codons.

	The First Site				The Second Site				The Third Site			
	T	C	A	G	T	C	A	G	T	C	A	G
*S. moschatus*	28.5	25.2	33.6	12.7	24.7	25.0	35.8	14.5	27.5	25.5	32.1	14.9
*S. moschatus isolate M11099*	28.3	25.5	33.4	12.8	24.4	25.3	35.6	14.7	27.7	25.5	32.1	14.8
*S. oweni*	30.3	23.6	34.1	12.1	27.1	23.8	35.1	14.0	28.6	24.2	32.7	14.5
*P. leucura*	28.0	25.8	33.2	13.0	23.8	25.8	35.3	15.0	27.5	25.5	32.1	15.0
*M. robusta*	31.0	22.7	34.6	11.7	27.3	22.1	37.5	13.0	29.0	23.8	33.5	13.8
*M. wogura*	30.2	23.5	34.4	11.9	26.4	23.0	37.3	13.2	28.6	24.2	33.2	14.0
*T. europaea*	28.6	24.6	33.9	12.9	24.8	24.2	36.0	15.0	27.5	25.0	32.4	15.0
*T. aquitania*	28.2	25.1	34.0	12.8	24.7	24.5	36.2	14.7	27.6	24.8	32.5	15.1
*T. occidentalis*	28.4	24.8	33.7	13.1	24.3	24.8	36.1	14.9	27.2	25.2	32.5	15.1
*C. cristata*	30.4	23.1	35.3	11.2	26.9	22.3	37.5	13.3	28.5	23.6	34.0	13.8
*U. talpoides*	30.2	23.6	34.2	12.0	26.4	23.9	35.8	13.9	28.3	24.6	32.9	14.2
*U. andersoni*	31.8	23.1	32.8	12.4	28.4	22.5	34.6	14.6	30.0	23.3	32.2	14.5
*U. gracilis*	32.1	23.0	32.4	12.5	28.9	22.1	35.2	13.9	30.0	23.0	32.5	14.5
*U. soricipes*	32.3	22.4	33.2	12.0	29.0	21.9	35.6	13.4	30.2	22.7	32.8	14.2

**Table 3:** Comparison of initiation/termination codons of 13 protein-coding genes.

	*ND1*	*ND2*	*COX1*	*COX2*	*ATP8*	*ATP6*	*COX3*	*ND3*	*ND4L*	*ND4*	*ND5*	*ND6*	*Cyt b*
	Initiation codons												
	Termination codons												
*S. moschatus*	ATG TA -	ATA T- -	ATG TAA	ATG TAA	ATG TAA	ATG TAA	ATG T- -	ATT T- -	ATG TAA	ATG T- -	ATA TAA	ATG TAA	ATG AGA
*S.moschatus* *Isolate M11099*	ATG TA -	ATA T- -	ATG TAA	ATG TAA	ATG TAA	ATG TAA	ATG T- -	ATT T- -	ATG TAA	ATG T- -	ATT TAA	TTA CAT	ATG AGA
*S. oweni*	ATG TA -	ATT T- -	ATG TAA	ATG TAA	ATG TAA	ATG TAA	ATG T- -	ATT T- -	ATG TAA	GTG T- -	ATA TAA	ATG TAA	ATG AGA
*P. leucura*	ATG TA -	ATA T- -	ATG TAA	ATG TAA	ATG TAA	ATG TAA	ATG T- -	ATT T- -	ATG TAA	ATG T- -	ATT TAA	ATA TAA	ATG AGA
*M. robusta*	ATG TA -	ATA T- -	ATG TAA	ATG TAA	ATG TAA	ATG TAA	ATG T- -	ATT T- -	ATG TAA	ATG T- -	ATT TAA	ATG TAA	ATG AGA
*M. wogura*	ATG TAA	ATA TAG	ATG TAA	ATG TAA	ATG TAA	ATG TAA	ATG T- -	ATT T- -	ATG TAA	ATG T- -	ATT TAA	ATG TAA	ATG AGA
*T. europaea*	ATG TAA	ATA TAG	ATG T- -	ATG TAA	ATG TAA	ATG TAA	ATG T- -	ATT T- -	ATG TAA	ATG T- -	ATT TAA	ATG TAA	ATG T- -
*T. aquitania*	ATG TAA	ATA T- -	ATG TAA	ATG TAA	ATG TAA	ATG TAA	ATG T- -	ATT T- -	ATG TAA	ATG T- -	ATT TAA	ATG TAA	ATG AGA
*T. occidentalis*	ATG TAA	ATA T- -	ATG TAA	ATG TAA	ATG TAA	ATG TAA	ATG T- -	ATT T- -	ATT TAA	ATG T- -	ATT TAA	ATG TAA	ATG AGA
*C. cristata*	ATG T- -	ATA T- -	ATG TAA	ATG TAA	ATG TAA	ATG TAA	ATG T- -	ATT T- -	ATG TAA	ATG T- -	ATT TAA	ATG TAA	ATT AGA
*U. talpoides*	ATG TAG	ATG TAG	ATG TAA	ATG TAA	ATG TAA	ATG TAA	ATG T- -	ATT T- -	ATG TAA	ATG T- -	ATT TAA	ATG TAA	ATG AGA
*U. andersoni*	ATG T- -	ATT T- -	ATG TAG	ATG TAA	ATG TAA	ATG TAA	ATG TA -	ATA T- -	ATG TAA	ATG T- -	ATT TAA	ATG TAA	ATG AGA
*U. gracilis*	ATG T- -	ATA T- -	ATG TAG	ATG TAA	ATG TAA	ATG TAA	ATG TA -	ATA T- -	ATG TAA	GTG TAA	ATT TAA	ATG TAA	ATG AGA
*U. soricipes*	ATG T- -	ATA T- -	ATG TAG	ATG TAA	ATG TAA	ATG TAA	ATG TA -	ATA T- -	ATG TAA	GTG T- -	ATT TAA	ATG TAA	ATG AGA

**Table 4:** Location of the central domain, extended termination associated sequences (ETAS-1, ETAS-2), and conserved sequence blocks (CSB-1, CSB-2, and CSB-3) in the control region of Talpidae animals’ mitochondrial genomes.

Organism	Central Domain	ETAS-1	ETAS-2	CSB-1	CSB-2	CSB 3
*S. moschatus*	344–660	180–238	257–327	523–548	885–902	937–955
*S. moschatus isolate M11099*	344–660	180–238	257–327	523–548	885–902	1002–1040
*S. oweni*	405–721	234–302	320–388	585–610		1067–1085
*P. leucura*	362–679	196–256	275–345	541–566	1046–1063	1096–1114
*M. robusta*	366–688	194–254	273–349	550–575	1064–1081	1115–1133
*M. wogura*	362–679	191–251	270–345	542–567	996–1013	1046–1064
*T. europaea*	349–668	179–237	255–332	529–554	1072–1089	1121–1139
*T. aquitania*	358–677	190–247	265–341	538–563	1013–1030	1062–1080
*T. occidentalis*	353–673	186–243	261–336	534–559	1146–1163	1195–1213
*C. cristata*	365–679	201–261	280–348	543–568	895–912	945–963
*U. talpoides*	342–658	180–238	256–325	522–547	1080–1097	1127–1145
*U. andersoni*	358–678	184–252	270–341	537–562	771–788	1043–1061
*U. gracilis*	369–684	214–283	302–380	595–620		
*U. soricipes*	376–690	201–269	287–358	554–579	807–824	1073–1090

**Table 5:** The sequence information of tandem repeats in the control region of Talpidae animals’ mitochondrial genomes.

	Period Size	Copy Number	Repeat Unit
*S. moschatus*	12	11	ACGTATACGCGC
*S. moschatus isolate M11099*	12	17	ATACACGCACGT
*S. oweni*	12	22	CGTATACACGCA
*P. leucura*	12	22	GTACGCACACAT
*M. robusta*	8	36	TACACGTA
*M. wogura*	8	27	ACACGTAT
*T. europaea*	16	20	CACAGGCGTATACACC
*T. aquitania*	10	23	TACGCACACG(A)
*T. occidentalis*	10	39	CACGTACGCA
*C. cristata*	8	12	ATACACGT
*U. talpoides*	10	31	CACACGTACG
*U. soricipes*	2	17	AT

## Data Availability

The genome sequence data that support the findings of this study is openly available in GenBank of NCBI at (https://www.ncbi.nlm.nih.gov/, accessed on 11 August 2021) under the accession number MZ594566.1.

## Supplementary Materials

The following supporting information can be downloaded at: https://www.mdpi.com/article/10.3390/ani13020186/s1. Figure S1: Sequence of control regions in the mitochondrial genome of Talpidae animals. The purple area indicates the extended termination sequence (ETAS-1, ETAS-2), the green area indicates the conserved sequence (CSB-1, CSB-2, CSB-3). Figure S2: Comparison of the mitochondrial *Cyt b* genes of 48 species belonging to the family Talpidae. Figure S3: Phylogenetic tree of 48 species belonging to the family Talpidae constructed by neighbor-joining method based on mitochondrial *Cyt b* genes. Figure S4: Phylogenetic tree of 48 species belonging to the family Talpidae constructed by minimum evolution method based on mitochondrial *Cyt b* genes. Table S1: Sequence information of the mitochondrial genome of 14 species belonging to the family Talpidae. Table S2: Sequence information of mitochondrial genome *Cyt b* genes of 48 species belonging to the family Talpidae. Table S3: Codons usage in the mitochondrial genome of Talpidae animals. Table S4: Genetic distance of Talpidae animals.
